# Effect of bilirubin and Gilbert syndrome on health: cohort analysis of observational, genetic, and Mendelian randomisation associations

**DOI:** 10.1136/bmjmed-2022-000467

**Published:** 2023-07-12

**Authors:** Fergus W Hamilton, KWM Abeysekera, Willie Hamilton, Nicholas J Timpson

**Affiliations:** 1 MRC Integrative Epidemiology Unit, Bristol, UK; 2 Infection Science, North Bristol NHS Trust, Bristol, UK; 3 Department of Liver Medicine, Bristol Royal Infirmary, Bristol, UK; 4 Medical School, University of Exeter, Exeter, UK

**Keywords:** genetics, gastrointestinal diseases, medicine

## Abstract

**Objectives:**

To compare associations between the Gilbert syndrome genotype in European populations, measured bilirubin concentrations, genetically predicted bilirubin using this genotype, and a wide range of health outcomes in a large cohort.

**Design:**

Cohort study including observational, genetic, and Mendelian randomisation analyses.

**Setting:**

22 centres across England, Scotland, and Wales in UK Biobank (2006-10), with replication in a national Finnish cohort (FinnGen).

**Participants:**

463 060 participants in the UK Biobank were successfully genotyped for a genetic variant (rs887829) that is strongly associated with Gilbert syndrome and 438 056 participants had measured bilirubin concentrations with linked electronic health record data coded using the tenth edition of the International Classification of Diseases. Replication analyses were performed in FinnGen (n=429 209) with linked electronic health record data.

**Main outcome measures:**

Odds ratios for the association between serum bilirubin concentrations, rs887829-T homozygosity (the risk genotype for Gilbert syndrome), genetically predicted bilirubin using rs887829-T allele carriage alone, and a wide range of health outcomes recorded in primary and secondary care.

**Results:**

46 189 participants in UK Biobank (about 10%) were homozygous for rs887829-T defining them as having the genotype characterising Gilbert syndrome. However, only 1701 (3%) of this group had a coded diagnosis of Gilbert syndrome. Variation at this locus explained 37.1% of all variation in measured serum bilirubin. In the observational analyses, higher bilirubin concentrations had strong inverse associations with a wide range of outcomes including overall health status, chronic obstructive pulmonary disease, myocardial infarction, and cholesterol measures. These associations were not identified in people with the Gilbert genotype. We identified associations with genetically predicted bilirubin concentrations and biliary and liver pathology (eg, odds ratio for cholelithiasis 1.16 (95% confidence interval 1.12 to 1.20); P=5.7×10^-16^) and a novel association with pityriasis rosea (1.47 (1.27 to 1.69), P=1.28×10^-7^).

**Conclusions:**

Only 3% of participants who are homozygous for rs887829-T have a recorded diagnosis of Gilbert syndrome. Carriers of this genotype have modest increases in the odds of developing biliary pathology and pityriasis rosea. Evidence from the analyses of genetic data suggests that bilirubin has no likely causal role in protection from cardiovascular disease, chronic obstructive pulmonary disease, or other key healthcare outcomes and therefore represents a poor target for therapeutic intervention for these outcomes.

**WHAT IS ALREADY KNOWN ON THIS TOPIC**

- Bilirubin is known to have strong antioxidant properties, and observational data suggest an inverse relationship with many health outcomes (eg, higher bilirubin is protective)
- Whether bilirubin is causal for these outcomes is uncertain
- Whether people who carry the genotype leading to Gilbert syndrome (a common cause of hyperbilirubinaemia) have different outcomes to people who do not carry this genotype is unknown

**WHAT THIS STUDY ADDS**

- In a large cohort study (n=463 060), around 10% of patients were homozygous for the Gilbert syndrome genotype
- Only 3% of participants who carry this genotype were diagnosed with Gilbert syndrome
- More participants with the Gilbert syndrome genotype had biliary pathology and pityriasis rosea, but no other health conditions

**HOW THIS STUDY MIGHT AFFECT RESEARCH, PRACTICE, OR POLICY**

- In contrast to the observational data, these results do not broadly support a causal role for bilirubin outside of biliary disease and pityriasis rosea
- Clinicians should be aware of the increased rates of biliary pathology in people with Gilbert syndrome, while researchers should not prioritise manipulating bilirubin as a therapeutic approach to the syndrome

## Introduction

Bilirubin is a breakdown product of haemoglobin and is widely measured as part of routine liver function testing. Bilirubin is known to have antioxidant properties and can reduce cellular exposure to reactive oxygen species in various in vitro and model animal systems.^1^ Additionally, observational studies have identified protective effects of increasing bilirubin concentrations on a wide range of outcomes.^2–5^ In particular, observational estimates have suggested large protective associations (eg, higher bilirubin concentrations were associated with lower odds) of myocardial infarctions and strokes.^3 5^

Despite these studies, the evidence that bilirubin is causal for these outcomes remains limited.^6 7^ Concentrations of bilirubin are known to be altered by overall health state, liver function, obesity, and lifestyle traits such as smoking and alcohol intake.^4^ In the context of decompensated cirrhosis, hyperbilirubinaemia reflects synthetic dysfunction, hepatic failure, and can also be nephrotoxic.^8^ Due to these associations, measured bilirubin concentrations are highly likely to be confounded by health state. An association between health state (eg, low body mass index) and both high bilirubin levels and outcomes of interest (eg, cardiovascular disease) means that reported observations might not be causal, and bilirubin merely reflects a marker of health state.

Gilbert syndrome is a common inherited condition association with mutations in the hepatic isoform (1A1) of uridine diphosphoglucose glucuronosyltransferase 1 (*UGT1A1*). This enzyme, produced in the liver, conjugates water insoluble unconjugated bilirubin to glucuronic acid. This conjugated bilirubin can then be excreted in the bile. Gilbert syndrome is associated with various mutations, but in European populations, homozygosity for a two base pair TA insertion into the promoter of the *UGT1A1* (rs3064744, also known as UGT1A*28) is the most common.^9^ This mutation impairs transcription of *UGT1A1* leading to around 20-30% of normal transcriptional values and a subsequent reduced amount of enzyme. This reduced enzyme function leads to higher concentrations of conjugated bilirubin. People who are homozygous for this allele have greatly reduced enzyme transcription and subsequently higher concentrations of conjugated bilirubin than people who are heterozygotes (ie, inheritance of Gilbert syndrome at this allele is generally considered as recessive). This insertion is in complete linkage disequilibrium with a common single nucleotide polymorphism (rs887829) that is commonly genotyped and well imputed, allowing genotyping at scale in large cohorts.^10^ This mutation is common (around 35% of Europeans are heterozygotes and 10% are heterozygotes). This variant alone explains around 30-40% of variation in serum bilirubin concentrations, and nearly all European people with Gilbert syndrome carry this genotype.^11 12^ Hereafter, we refer to rs887829-T homozygosity as the Gilbert syndrome genotype because we recognise that most people with this genotype will have elevated bilirubin but no formal diagnosis of Gilbert syndrome (ie, incomplete penetrance).

This locus has a strong association with bilirubin and consequently has a clear mechanism (impairing transcription of the enzyme). The enzyme is known to have no other function apart from breaking down bilirubin, providing the opportunity for Mendelian randomisation to be performed.^13^ In Mendelian randomisation, genetic variants (in this case the variant rs887829) are used as instrumental variables to explore the causal association between an exposure (in this case bilirubin) and outcomes (in this case a variety of health outcomes).^13^ With specific assumptions, Mendelian randomisation can provide an estimate of the causal association between an exposure and outcomes, which are often intractable in observational studies due to the presence of unmeasured confounding.^13^

In this study, we aimed to estimate the causal association between bilirubin and health outcomes using the Gilbert syndrome genotype as an instrumental variable and compare results with observational, measured analyses in a large UK volunteer cohort.

## Methods

### Study design

Our study is an observational, genetic, and Mendelian randomisation analysis of the effect of bilirubin on health outcomes, and an analyses of the risk and penetrance of Gilbert syndrome associated with the Gilbert syndrome genotype. We report this study in line with the STREGA^14^ guidance (online supplemental file S1).

10.1136/bmjmed-2022-000467.supp3 Supplementary data

### Setting

Participants in UK Biobank, a large volunteer cohort of about 500 000 people aged 5070 years, recruited across 22 UK centres, who have blood tests at recruitment and a large range of linked health data.^15^ For this study, we included all available participants for the observational study on the association between bilirubin concentration and health outcomes and included participants of European ancestry (defined below) for the genetic analyses.

### Exposures

On recruitment to UK Biobank, all participants had a sample taken for measurement of bilirubin and for genotyping as part of their initial assessment.^15^ Detailed description of the recruitment procedures and technical procedures are available elsewhere.^15^ In this analysis, we only used total bilirubin concentration because correlation between other markers (eg, unconjugated bilirubin) was very high (Pearson’s R=0.9), and this result is commonly reported in routine testing. The level of serum bilirubin was extracted directly from UK Biobank and analysed without adjustment or transformation.

For analysis of genetic data, we used the single nucleotide polymorphism rs887829, which is associated with nearly all cases of Gilbert syndrome in Europe and explains 30-40% of variation in bilirubin levels.^12^ We used in-house quality controlled genetic data for European ancestry participants.^16^ The data release for this analysis contained the cohort of successfully genotyped samples (n=488 377). By use of the UK BiLEVE array and 438 398 using the UK Biobank axiom array, 49 979 individuals were genotyped. Pre-imputation quality control, phasing, and imputation are described elsewhere;^15^ we describe post imputation quality control here.^16^

rs887829 was directly genotyped on both arrays used in UK Biobank. We only included participants with European ancestry as defined by an in-house k-means cluster analysis performed using the first four principal components provided by UK Biobank in the statistical software environment R.^16^ Our current analysis includes the largest cluster from this analysis (n=464 708).

We defined genetic exposure in multiple ways. Firstly, we performed an additive model, defining exposure as the number of Gilbert syndrome risk alleles (rs887829-T). Secondly, we analysed people who had a Gilbert syndrome genotype (eg, homozygotes for rs887829-T). Additionally, we performed a genetically predicted bilirubin model to maximise power and generate estimates on the same scale as the observational data (eg, µmol/L) for direct comparison and because neither the additive model nor the model comparing Gilbert syndrome genotypes to others explained all the variance in bilirubin concentration at this locus. In this model, the mean bilirubin for each genotype (CC, CT, and TT) was calculated, and each participant was assigned this value as their genetically predicted bilirubin.

### Outcomes and analysis

We extracted data for body mass index, C reactive protein, diabetes, renal function (using creatinine), presence of liver disease, and history of cancer, and for smoking and alcohol intake history. We also extracted the calculated Townsend deprivation index from UK Biobank. All of these data were collected via questionnaires at recruitment to UK Biobank (or for the biological traits, were measured at recruitment).^15^

Outcomes were analysed using a Phenome Wide Association Study approach (known as PheWAS) to classify and generate health outcomes in this study, using PHESANT.^17^ PHESANT extracts all health related codes from the tenth edition of the International Classification of Diseases (ICD-10), data from questionnaires, and all continuous variables from UK Biobank. PHESANT then performs appropriate transformations on the data, and tests the association between the exposure and the outcome. In total, 13 117 outcomes were available for inclusion. Health outcomes were derived from self-report or linked electronic health record data, whereas most biological and questionnaire data were recorded at recruitment or at subsequent follow-up visits at clinics. A detailed description of the data included in UK Biobank is available at the UK Biobank data showcase, with extensive literature on variable definitions.^15^ We used linear regression for continuous outcomes, logistic regression for binary traits, and multinomial regression for categorical outcomes. Variable definitions and choice of model are detailed in the original PHESANT paper.^17^ We excluded binary outcomes if fewer than 50 cases were available. For this study, we focused on reporting the ICD-10 coded health outcomes.

For our observational analyses on measured bilirubin, univariable logistic or linear regression was used to assess the association between bilirubin and outcome. A second and adjusted model was used including age, sex, smoking, and history of alcohol intake on recruitment, body mass index on recruitment, presence of liver disease, C-reactive protein, and level of deprivation. This model did not aim to capture all relevant confounding variables but represents an attempt to model whether adjusting for known large confounders of bilirubin alter the observational associations.

For our genetic model, we did not include covariates except genotyping chip, recruitment centre, and the first 10 principal components of the genetic data, with details of the derivation of these principal components provided elsewhere.^16^

Analyses was performed in R 4.0.4, with data management using the tidyverse package.^18^ PHESANT (https://github.com/MRCIEU/PHESANT) was used to process and generate health outcomes for testing.^17^

### Parallel analysis

To follow up our genetic findings, we re-analysed associations that met a threshold of P<0.05 in the analysis of genetically predicted bilirubin using a set of results from genome wide association studies (GWAS) from the FinnGen consortium.^19^ This consortium has performed and released GWAS summary statistics for numerous ICD-10 codes using an additive model.^19^ We extracted effect estimates for rs887829 from each GWAS.^19^ These estimates were then compared and meta-analysed with estimates from our additive model, which is on the same scale as the FinnGen data.

### Colocalisation

As linkage disequilibrium across this region is substantial, shared genetic signals that we identify might represent different causal variants (ie, another variant is near to rs887829 that is causal for the outcome, confounding our association). Therefore, for any genetic associations that we identified, we performed colocalisation^20^ using a prior GWAS of bilirubin performed by the Pan Pan-UK Biobank team. This GWAS is available via the IEU OpenGWAS database (ID: ukb-d-30840_irnt). For each identified outcome, we identified the largest GWAS in European populations from the IEU OpenGWAS database. A region 70 000 kilobases either side of the top GWAS hit (rs887829) was extracted from each GWAS and then colocalisation was done using the coloc package in R.

### Sex specific analyses

Bilirubin metabolism is known to differ by biological sex. We therefore re-ran all genetic analyses in men and women separately to identify sex specific effects. Heterogeneity between effects was calculated using the meta package in R.

### Smoking status

The protective effect of bilirubin could plausibly occur only in people who are exposed to oxidative stress. Smoking is a major cause of oxidative stress, and previous studies have suggested a protective effect of bilirubin only in people who smoke.^21^ Therefore, as a secondary analysis, we performed analyses stratified by smoking status (never, ex, or current) on recruitment to UK Biobank.

### Multiple testing

We aimed to test a large number of associations, therefore, some associations would be due to chance. As such, we report the actual P value and a false discovery rate corrected P value at 5% in the tables, and we report the actual P value in the text. We recognise that false discovery rate correction may not be appropriate given the correlation between outcomes, hence reporting the raw P value, but we provide a false discovery rate correction for readers who want some correction for multiple testing.

### Sample size and power

We calculated estimated power for our genetic analyses using a recognised approach.^22^ We fixed the number of controls at 450 000, set an alpha of 0.05 and set the explanatory power of the instrumental variable to be 37%, based on our results. Estimated power given various assumptions is shown in online supplemental figure S1. We estimated a power of more than 80% to detect an odds ratio of more than 1.25 for 77% of outcomes reported in our primary analysis (those with >334 cases).

10.1136/bmjmed-2022-000467.supp1 Supplementary data

### Patient and public involvement

Patients and the public were not involved in this study. We do not have plans to disseminate our results to research participants. Our study results will be return to UK Biobank in line with their policy.

## Results

### Demographics of measured phenotypic and genetic associations with bilirubin

In total, 438 056 participants had an available bilirubin result and could be included in the phenotypic analyses. Serum bilirubin was approximately log normally distributed, with a median result of 8.1 µmol/L (interquartile range 6.5-10.4). Table 1 describes the sample for each quarter of recorded value. In line with previous studies, strong associations were noted between bilirubin and various relevant outcomes including body mass index, age, sex, alcohol intake, smoking, renal function, and liver disease (all P<0.001). The most striking associations were with sex and smoking status: people in the lowest quarter were 75% female and 15% were current smokers, whereas people in the highest quarter were 35% female and 7% were current smokers.

**Table 1:** Characteristics on recruitment to the UK Biobank stratified by serum bilirubin level

Characteristic	Quarter 1 (n=109 514)	Quarter 2 (n=109 514)	Quarter 3 (n=109 514)	Quarter 4 (n=109 514)
Serum bilirubin, µmol/L (interquartile range)	5.5 (4.9 to 6.0)	7.2 (6.8 to 7.7)	9.1 (8.6 to 9.7)	12.9 (11.4 to 16.1)
Risk alleles for Gilbert syndrome:				
None	73 977 (68)	60 913 (56)	47 903 (44)	21 874 (20)
One (heterozygote)	34 920 (32)	47 065 (43)	57 205 (52)	50 458 (46)
Two (homozygote)	617 (1)	1536 (1)	4406 (4)	37 182 (34)
Sex:				
Female	82 487 (75)	66 031 (60)	50 355 (46)	38 653 (35)
Male	27 027 (25)	43 483 (40)	59 159 (54)	70 861 (65)
Age on recruitment	58 (51 to 64)	59 (51 to 64)	59 (51 to 64)	58 (50 to 64)
Townsend deprivation index	−2.05 (−3.58 to 0.67)	−2.26 (−3.69 to 0.25)	−2.33 (−3.73 to 0.13)	−2.38 (−3.77 to 0.03)
Unknown	121	126	127	139
Alcohol intake frequency:				
Daily or almost daily	17 068 (16)	22 358 (20)	25 821 (24)	26 942 (25)
Three or four times a week	21 877 (20)	25 483 (23)	28 063 (26)	28 912 (26)
Once or twice a week	29 340 (27)	29 076 (27)	28 331 (26)	28 171 (26)
One to three times a month	14 667 (13)	12 642 (12)	11 109 (10)	10 435 (10)
Special occasions only	16 345 (15)	12 178 (11)	9861 (9)	9046 (8)
Never	10 032 (9)	7623 (7)	6213 (6)	5867 (5)
Prefer not to answer	116 (0.1)	87 (<0.1)	65 (<0.1)	88 (<0.1)
Smoking status:				
Current	16 153 (15)	11 946 (11)	9859 (9.0)	7761 (7)
Previous	36 223 (33)	38 916 (36)	40 157 (37)	40 096 (37)
Never	56 622 (52)	58 190 (53)	59 065 (54)	61 230 (56)
Prefer not to answer	447 (<1)	395 (<1)	382 (<1)	374 (<1)
Body mass index	27.1 (24.3 to 30.6)	26.7 (24.1 to 29.9)	26.6 (24.1 to 29.6)	26.5 (24.0 to 29.4)
Creatinine (µmol/L)	66 (58 to 75)	69 (61 to 79)	72 (63 to 82)	75 (66 to 84)
History of any liver disease	2909 (3)	2632 (2)	2763 (3)	3245 (3)

Data are number (percentage), unless otherwise specified. All P values for comparison were <0.001. Not all characteristics were available for all participants.

Gilbert syndrome’s genotype (rs887829-T allele carriage) and measured serum bilirubin showed a strong association: 34% of participants in the top quarter of serum bilirubin were homozygotes for the risk allele and fewer than 1% of participants in the bottom quarter were homozygotes.

Consistent with this, genetic variation at the Gilbert syndrome locus explained a large proportion of changes in observed bilirubin concentrations. People who were heterozygotes or homozygotes at rs887829-T had higher bilirubin concentrations than people who were not carriers (median serum bilirubin was 15.7 μmol/L for homozygous alleles; 8.5 μmol/L for heterozygous alleles; and 7.2 μmol/L for no Gilbert syndrome risk alleles). This effect appeared to be non-additive, with larger effects seen in people with homozygous risk alleles (table 2). In a genotypic model, variation at rs887829 alone explained 37.1% of all variation in serum bilirubin. Unlike measured bilirubin, variation at rs887829 was not associated with health outcomes at baseline recruitment; although, weak evidence suggested a decreased alcohol intake in those with no copies of rs887829-T and marginally higher levels of liver disease in comparison to people with either hetereozygous or homozygous risk alleles (table 2).

**Table 2:** Associations with Gilbert syndrome risk alleles and demographic variables on recruitment to UK Biobank

Characteristic	No Gilbert syndrome risk alleles (n=216 555)	Heterozygotes for Gilbert syndrome risk allele (n=200 316)	Homozygotes for Gilbert syndrome risk allele (n=46 189)	P value*
Serum bilirubin (µmol/L)	7.2 (5.9 to 8.8)	8.5 (6.9 to 10.6)	15.7 (12.0 to 20.4)	<0.001
Sex:				0.7
Female	117 140 (54)	108 116 (54)	24 992 (54)	
Male	98 591 (46)	91 449 (46)	21 041 (46)	
Age on entry to UK Biobank (years)	59 (51 to 64)	59 (51 to 64)	59 (51 to 64)	0.8
Townsend deprivation index	–2.25 (–3.70 to 0.27)	−2.26 (−3.70 to 0.28)	−2.25 (–3.68 to 0.30)	0.4
Alcohol intake frequency:				0.004
Daily or almost daily	45 620 (21)	42 024 (21)	9375 (20)	
Never	14 673 (6.8)	13 428 (6.7)	3261 (7.1)	
Once or twice a week	56 518 (26)	52 435 (26)	12 110 (26)	
One to three times a month	24 069 (11)	22 124 (11)	5278 (11)	
Prefer not to answer	186 (<0.1)	155 (<0.1)	28 (<0.1)	
Special occasions only	23 184 (11)	21 702 (11)	5029 (11)	
Three or four times a week	51 354 (24)	47 584 (24)	10 930 (24)	
Smoking status:				0.7
Current	22 526 (10)	20 835 (10)	4849 (11)	
Never	116 001 (54)	106 870 (54)	24 759 (54)	
Prefer not to answer	807 (0.4)	721 (0.4)	162 (0.4)	
Previous	76 270 (35)	71 027 (36)	16 241 (35)	
Body mass index (kg/m^2^)	26.7 (24.1 to 29.9)	26.7 (24.1 to 29.9)	26.7 (24.1 to 29.9)	0.8
Creatinine (µmol/L)	70 (61 to 81)	70 (61 to 81)	70 (62 to 81)	0.11
History of liver disease	5596 (2.6)	5311 (2.7)	1332 (2.9%)	0.001

Data are numerator (percentage) or median (interquartile range), unless otherwise specified. *Pearson’s χ² test; Kruskal-Wallis rank sum test.

### Associations of rs887829-T homozygosity (Gilbert syndrome)

**Figure 1:**
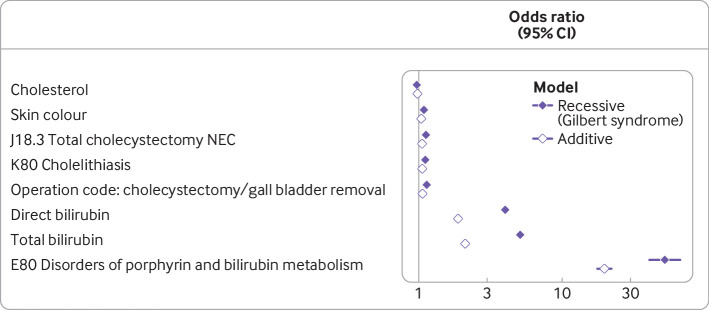
Associations between variation at rs887829 and questionnaire and health outcomes in UK Biobank. The blue points reflect the association between homozygotes (Gilbert syndrome genotype) and each outcome, whereas the white points reflect the association between each additional T allele at this locus. X axis is on a log scale. NEC=not elsewhere classified

We explored clinical associations with rs887829-T homozygotes (Gilbert syndrome), testing approximately 13 000 outcomes from questionnaire data, electronic health records, and anthropometric data, although we focus this paper on the healthcare coded outcomes (online supplemental table 1, figure 1). We report raw P values in the text, but present raw and false discovery rate adjusted P values in the supplement. rs887829-T homozygosity was associated with a clinical diagnosis of Gilbert syndrome (odds ratio 48.6 (95% confidence interval 42.7 to 55.5), P<1×10^-320^); however, only 1701 (~3%) of 46 089 patients had a formal diagnosis of Gilbert syndrome recorded.

10.1136/bmjmed-2022-000467.supp2 Supplementary data

Patients homozygous for rs887829-T had increased rates of disease related to the gallbladder and associated healthcare use compared with those with one or no allele, with higher rates of gallstones (odds ratio 1.16 (95% confidence interval 1.12 to 1.20), P=5.7×10^-16^), cholecystectomy (1.19 (1.14 to 1.25), P=2.6×10^-14^), and associated procedures. For example, odds were higher for endoscopic retrograde cholangiopancreatography (1.42 (1.21 to 1.66), P=1.22 × 10^-5^) and for complications such as cholecystitis (1.17 (1.07 to 1.26); P=1.5×10^-4^).

Other associations included skin colour and associated questionnaire outcomes (eg, ease of skin tanning), both of which had strong associations (P<1×10^-7^), and the skin condition pityriasis rosea (1.47 (1.27 to 1.69), P=1.28×10^-7^), which is not a known association of Gilbert.

rs887829-T homozygosity was associated with small changes in specific measured biomarkers (calcium, cholesterol, and HbA_1c_), although the absolute effect size of these changes was small: standard deviation change in calcium −0.037 (95% confidence interval −0.027 to −0.047), cholesterol 0.023 (0.012 to 0.033), and Hba_1c_ −0.019 (−0.01 to −0.032).

### Association between bilirubin, genetically predicted bilirubin, and associations with health outcomes

Given the above findings, which suggested a relatively weak association between Gilbert syndrome and major health outcomes, we investigated the association between (observational) measured bilirubin levels and health outcomes and compared this relation with genetic data.

In both unadjusted and adjusted analyses (online supplemental tables 2 and 3), serum bilirubin had a strong relation with a wide range of health outcomes. Of the 839 health outcomes included, 418 (50%) showed evidence of association at an unadjusted threshold of P<0.05. When adjusting for age, sex, smoking, alcohol, liver disease, baseline C-reactive protein, body mass index, and renal function, 277 (66%) of these 418 outcomes persisted at this threshold. For nearly all associations (373 (89%) of 418), the effect estimate for the unadjusted model was greater than the adjusted model. The strongest association was with ICD code E80 for disorders of porphyrin and bilirubin metabolism (unadjusted odds ratio 1.19 (95% confidence interval 1.17 to 1.21), P<1×10^–320^) per each mmol/L increase in bilirubin), which represents the code for Gilbert syndrome. Some associations had very strong statistical evidence but had implausible causal mechanisms, for example, an association with increased rates of perineal laceration during delivery (P=1.8×10^–30^).

**Figure 2:**
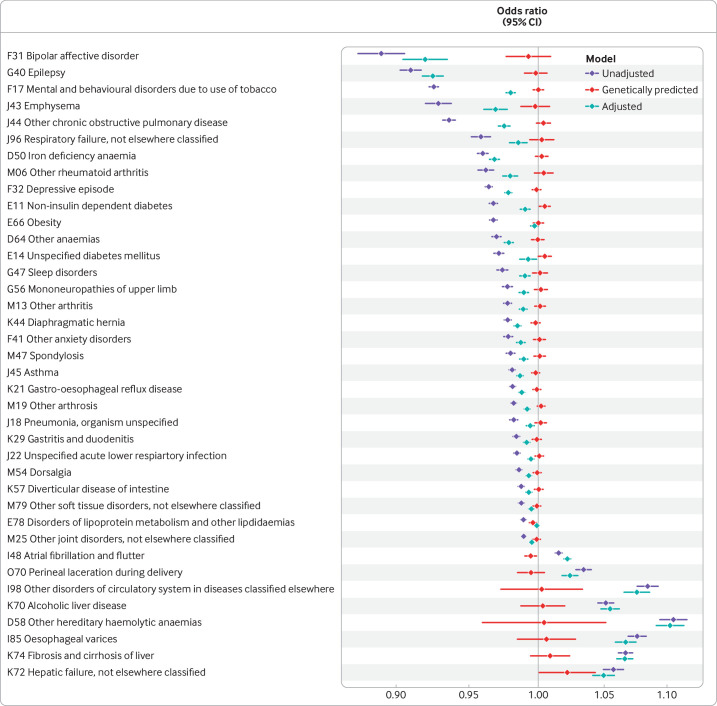
Associations between bilirubin (on a scale of µmol/L) and various health outcomes in logistic regression. The top 38 unadjusted associations are shown. The blue bars represent the measured bilirubin, unadjusted for covariates. The red bars represent genetically predicted bilirubin using variation at rs887829 only. The green bars represent the effect of bilirubin adjusted for multiple covariates (see text and methods)

By contrast, evidence of association in genetic analyses of Gilbert syndrome was not abundant (online supplemental tables 4 and 5, figure 2). For example, decreased serum bilirubin was associated with protection from bipolar disorder (odds ratio 0.89 (95% confidence interval 0.87 to 0.90), P=1.6×10^-38^), an association that only reduces slightly with adjustment for covariates (0.92 (0.90 to 0.94), P=1.5×10^-22^). However, the association with genetically predicted bilirubin was null (0.99 (0.98 to 1.01), P=0.40).

**Figure 3:**
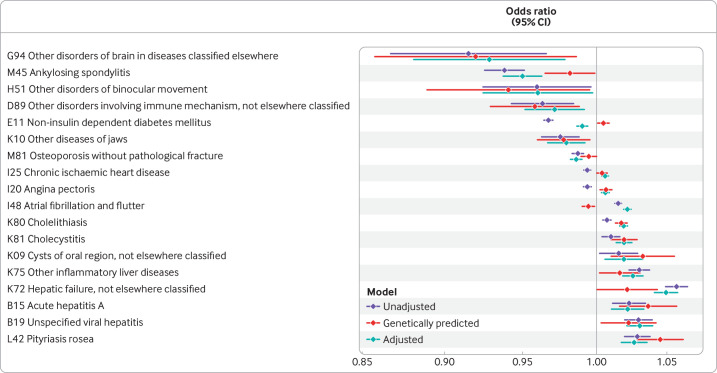
Associations of bilirubin that had a P value of <0.05 in logistic regression across unadjusted models, covariate adjusted, and genetic models for the association between bilirubin and outcome. The blue bars represent the measured bilirubin, unadjusted for covariates. The red bars represent genetically predicted bilirubin. The green bars represent the effect of bilirubin adjusted for multiple covariates (see text and methods). Scale is per μmol/L

In total, 18 outcomes had associations at a P value threshold of 0.05 in adjusted, unadjusted, and genetically predicted model (figure 3). In four of these models, the directionality was not consistent, leaving 14 outcomes with suggestive evidence of an association. Four of these were liver diseases (hepatitis A, unspecified viral hepatitis, other inflammatory liver diseases, and unclassified hepatic failure), while two were the previously identified associations with gallbladder pathology (cholelithiasis and cholecystitis). The other associations included increased odds of pityriasis rosea with increasing bilirubin concentrations, and decreased odds of ankylosing spondylitis and osteoporosis.

As we were testing a wide range of health conditions, many of these associations are likely due to chance. To further test whether these associations might be real, we attempted to replicate the analysis by identifying effect estimates for the same ICD-10 code from FinnGen.

**Figure 4:**
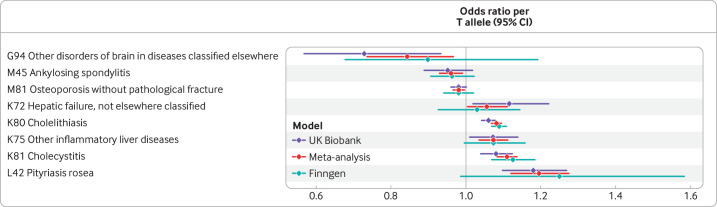
Nominally significant (P<0.05) associations between rs887829-T allele presence and outcomes in UK Biobank, FinnGen, and in meta-analysis. Odds ratios are presented per each additional T allele

We were able to identify 14 matching conditions and when combining effects across studies in meta-analysis across FinnGen and UK Biobank, eight of these persisted (online supplemental table 6, figure 4). These included the two direct associations of biliary disease (cholecystitis and cholelithiasis), two liver conditions (other hepatic failure and other inflammatory liver disease), an increased odds of pityriasis rosea, and a decreased odds of osteoporosis, ankylosing spondylitis, and so-called other disorders of brain. The strongest evidence outside of biliary disease was that for pityriasis rosea, with an odds ratio of 1.05 ((95% confidence interval 1.03 to 1.06), P=8 × 10^–8^) per each µmol/L increase in bilirubin, a potentially clinically relevant effect size.

### Sensitivity analyses

Colocalisation analyses are reported fully in the online supplemental results. For traits that were associated with the Gilbert syndrome genotype, we identified the largest available GWAS in European populations. For all outcomes except pityriasis rosea, colocalisation suggested a reasonable (posterior probability >0.5) probability of a shared causal variant. For pityriasis rosea, the posterior probability was 0.15, but this possibly reflected the low number of cases in the pityriasis rosea GWAS (n=134) and was sensitive to prior probability assumptions.

Effect estimates could plausibly differ in people exposed to high levels of oxidative activity, and evidence suggests differences in serum bilirubin in smokers, therefore, we re-ran analyses stratifying by smoking status (current smoker (n=48 243), ex-smoker (n=163 586); never smoker n=247 668, online supplemental tables 7–9). As we required a minimum number of 200 cases for each outcome, we report a differing number of outcomes for each model, which we performed on all outcomes (n~12 000). We then meta-analysed estimates for ICD-10 coded health outcomes from the three groups to identify heterogeneity (online supplemental table 7). We found little evidence for heterogeneity in effect with no single outcome having an overall effect and meeting a strict cut-off for heterogeneity (P<0.01).

We re-ran our genetic analyses using the Gilbert genotype in men and women separately. In general, effect estimates in men and women were similar, although they were slightly larger in men. Online supplemental figure S2 shows effect estimates for men and women for all conditions where we identified a nominal (P<0.05) sex combined association. This effect was most pronounced for pityriasis rosea, where the odds ratio in men was 1.96 (95% confidence interval 1.60 to 2.39), but in women was 1.15 (0.93 to 1.41). Thirty five conditions met a nominal test of heterogeneity (P<0.05) in effects between men and women and are visualised in online supplemental figure S3. Given the number of tests performed, many of these are likely to represent chance rather than a true sex-by-genotype interaction. These results are fully reported in online supplemental tables 10 and 11.

## Discussion

In this large volunteer cohort of European adults, 10% of participants were homozygous for rs887829-T (Gilbert syndrome genotype) and 40% of participants were heterozygous. This allele had a large and non-additive effect on serum bilirubin, with 37% of all variances in bilirubin explained by variation at this locus alone. Fewer than 4% of participants who were homozygous for rs887829-T had a diagnosis of Gilbert syndrome recorded, reflecting the largely benign nature of the condition and the incomplete penetrance at this locus.

In observational analyses, increased serum bilirubin was associated with (and largely protective) for a large range of health outcomes and anthropometric traits, although potential confounders (smoking, alcohol, comorbidities) were associated, adding to the challenge of causally interpreting effect estimates. In contrast to the observational data, patients with the Gilbert syndrome genotype had few associations with any healthcare outcomes; although we did identify increased associations with gallbladder pathology, which presumably relates to a direct increase in pigmented biliary stone formation secondary to increased unconjugated bilirubin. Additionally, we identified a small increased odds of some liver conditions, although these could relate to either miscoding of Gilbert syndrome or are more readily diagnosed in those with Gilbert syndrome. The other association we identified is, to the best of our knowledge, a previously unreported association with the skin condition pityriasis rosea. This condition—a common fine rash that occurs in response to various insults, including infection—had a robust association with Gilbert in both people who were heterozygous or homozygous at increased risk. This association warrants further investigation and seems unlikely to be simply related to the change in skin tone noted with some infections in patients with Gilbert syndrome. We additionally identified larger effect estimates in men than in women for pityriasis rosea, a further topic worthy of future research.

In subsequent analyses, we showed the large difference between observational and genetic estimates and confirmed genetic associations in a second, independent, dataset (FinnGen). We identified the increased odds of pityriasis rosea with increased bilirubin, and identified suggestive protective effects (decreasing odds with increasing bilirubin) of bilirubin on the odds of ankylosing spondylitis and osteoporosis. For the association with ankylosing spondylitis, a previous study identified differences in bilirubin between cases and controls. However, we would caution over-interpretation of these data; the effect estimates were small and close to the null despite the large sample size in our study and the prior study's probability of an association remains small.^23^

Finally, we compared estimates in current smokers, ex-smokers, and never smokers, in line with previous literature suggesting a potential interaction with Gilbert syndrome and smoking status.^21^ In contrast to these reports, we did not identify strong evidence of heterogeneity of effect, which supports our overall analyses.

### Strengths and limitations

This study has a large overall sample size (n~450 000), high quality genotyping at the locus of interest, and linked blood tests and electronic health records. Additionally, we were able to replicate our genetic associations in a large, independent dataset. Due to these factors, the precision and reliability of our reported associations is strong.

However, our study has limitations. In particular, we rely on self-report and healthcare coding data for our outcomes, both of which are likely to have some inaccuracies, and are likely to be under-reports of true outcome incidence. Although for most instances these occurrences are unlikely to lead to bias (because inaccuracies are not correlated with Gilbert syndrome genotype), this might explain some of the associations we see with the liver related outcomes. For example, clinicians and patients are more likely to seek referral or diagnosis for any liver condition if the bilirubin concentration is higher than normal, which is likely to lead to differential case ascertainment in those with Gilbert syndrome. This effect is shown by higher rates of endoscopic retrograde cholangiopancreatography (odds ratio 1.42 (95% confidence interval 1.21 to 1.66)) in people who carry the Gilbert syndrome genotype, which probably reflects increased clinician testing due to increased bilirubin concentrations because this effect is greater in size than that on biliary disease for example (odds ratio for gallstones 1.16 (1.12 to 1.20).

This may explain some of the increased rates of liver diseases, especially as bilirubin is often used as a diagnostic test to stratify or intervene in suspected hepatobiliary disease. As effect estimates for many of these conditions are small (odds ratio <1.1), the increased odds may be all due to differential case ascertainment. Against this, conditions such as gallstones (diagnosed often by abdominal imaging) are less susceptible to this bias, and increased rates of gallstones in those with higher bilirubin is biologically plausible. Additionally, although we have a large cohort, absolute number of cases of certain outcomes are low, and preclude firm conclusions. For approximately 80% of the included outcomes, we are powered to identify moderate size effects (odds ratio >1.25), but for those with lower case counts we are necessarily less confident on the precision of our estimates. Given the interconnectedness of biology, however, we would suggest that our summary results do not support a broadly causal role of bilirubin in the development of health conditions outside of the biliary tract and of our novel association with pityriasis rosea, in stark contrast to the associations identified in observational data.

In this analysis, because of the complexity of applying non-linear approaches, we used linear (or generalised linear, for example, logistic) regression models to assess the genetic associations. Bilirubin could possibly be truly causal for some of our outcomes that we have defined as null because we have not captured this non-linearity. However, this association would require a very specific relation that would have no overall effect in a linear model (eg, a completely U-shaped model), which is biologically implausible for many of our outcomes, and our approach would capture some non-linear effects (eg, those with a monotonic risk increase but that simply differed in shape).

As with any Mendelian randomisation study, a risk of horizontal pleiotropy from the instruments is possible. This effect occurs when the genetic instrument is acting not through the exposure (eg, our variant alters the risk of each outcome not through its effect on bilirubin).

Although possible, this effect is unlikely given the location of the variant and known function of both the gene. Additionally, given our null results for most outcomes, this either suggests a null pleiotropic effect, or an equal and opposing pleiotropic effect that balances out the direct effect through bilirubin. This is implausible for most outcomes, but possible for some.

Finally our data largely comes from UK Biobank, which is known to be non-representative of the whole UK population, with more than r than re than 94% of the cohort of European ancestry and on average healthier on many metrics, so our effect estimates may not be entirely accurate when transferred to the whole population.^24^ The effects of selection bias on effect estimates in genetic studies is an area of active research and has the potential to lead to false positive (and false negative associations).^25 26^ However, we would not expect an interaction between Gilbert syndrome genotype and selection into UK Biobank, and the relative risk of these conditions should remain the same within UK Biobank.

### Comparisons with previous literature

Much previous literature has reviewed the observational data linking bilirubin to various health outcomes (eg, systematic reviews in stroke,^5^ myocardial infarction,^3^ diabetes complications,^27^ all cause mortality,^4^ and chronic obstructive pulmonary disease^2^). Nearly all of this literature has identified a protective association, which is in line with our data. Additionally, a some genetic studies have investigated a range of outcomes, along with either the Gilbert syndrome risk locus or other genetic variants associated with bilirubin levels.^6 21 28–35^ In the most similar study, Stender and colleagues genotyped participants in Copenhagen for the Gilbert syndrome risk locus, and identified a null association with ischaemic heart disease,^6^ but did identify an association with gallstone disease.^7^ In a similar approach, Knutsor and colleagues genotyped 3989 participants for the same locus and again identified a null result.^29^ By contrast, papers using other genetic instruments (eg, variants in *SLCO1B1*)^21 32^ or a polygenic approach (combining multiple single nucleotide polymorphisms)^30 33^ found multiple associations (protective and harmful). We should not be surprised by these results, and as a result of our data, the previous associations are likely to represent chance or pleiotropy. Bilirubin is downstream of a large number of metabolic processes, and as such, most genetic variants that affect bilirubin (outside the Gilbert syndrome risk locus) are involved in other health processes (eg, cholesterol metabolism for *SLCO1B1*). Therefore unsurprisingly, these variants are associated with health outcomes, which then bias the genetic associations. By contrast, the Gilbert syndrome risk locus is well understood and the enzymes' main function is to breakdown bilirubin, limiting the risk of pleiotropy and providing an excellent locus to perform Mendelian randomisation.

### Implications

Our study provides a broad assessment of both Gilbert syndrome and the causal role of bilirubin in health. Contrary to previous reports of the protective nature of Gilbert syndrome, we identified few causal associations except a novel association with pityriasis rosea and an association with gallbladder pathology.

Despite a large sample size, no protective associations were identified. Previously reported associations in observational (and in some cases genetic) data are highly likely to be confounded to some extent. However, the novel association with pityriasis rosea deserves further investigation, and clinicians should be aware of the higher rates of biliary disease in people with Gilbert syndrome.

### Conclusions

Despite in vitro activity^1^ and strong observational associations, the application of Mendelian randomisation here suggests that life course exposure to altered bilirubin concentrations is unlikely to be causally related to a broad range of health outcomes. However, we did identify a clear association between people who carry the Gilbert syndrome genotype and higher rates of biliary disease, which clinicians should be aware of, and a novel association with pityriasis rosea, which deserves further investigation.

## Data Availability

All data relevant to the study are included in the article or uploaded as supplementary information. All results (and data needed to generate plots) are reported in the Supplementary Tables and code and the raw data are available to replicate the figures at https://github.com/gushamilton/gilberts. FinnGen associations at rs887829 are available via the FinnGen website.^36^
