# Supplementary material for: Effect of bilirubin and Gilbert syndrome on health: cohort analysis of observational, genetic, and Mendelian randomisation associations
Source: BMJ Med. 2023 Jul 12;2(1):e000467. doi: 10.1136/bmjmed-2022-000467 (PMC10347488; doi:10.1136/bmjmed-2022-000467)
Supplement: Supplementary data [file bmjmed-2022-000467supp003.pdf]

Supplementary Results: Colocalization

Colocalization is the process of identifying whether genetic signals in a region are likely to share the same causal variant.[1] It is most commonly performed as a Bayesian approach using summary statistics from Genome Wide Association Studies (GWAS) on differing outcomes. As a Bayesian approach, prior probabilities of e.g. a shared causal variant must be provided.

The posterior probability of multiple hypotheses are reported, alongside sensitivity analyses with differing prior probabilities. The hypotheses are:

- H_0: No association with either trait
- H_1: Association with trait 1, not with trait 2
- H_2: Association with trait 2, not with trait 1
- H_3: Association with trait 1 and trait 2, two independent SNPs
- H_4: Association with trait 1 and trait 2, one shared SNP

In this paper, we are most interested in the posterior probability of H_4, the probability that a single shared causal variant explains both the level of bilirubin and the outcome of interest. We therefore perform colocalization on multiple separate outcomes individually using the *coloc* package in R. Specifically, we use the *coloc.abf* function, and chose a region 70kb either side of the top GWAS hit for bilirubin, which is our instrumental variable (rs887829, position on GrCh37; 2:234668570). We extracted this region from a GWAS of inverse rank normal transformed bilirubin performed by the Pan-UKB team (IEU GWAS ID: ukb-d-30840_irnt, n = 342,829), which was performed in European ancestry participants in UK Biobank.[2] Details of each outcome GWAS are in Table 1. All were performed in UK Biobank except the GWAS for pityriasis rosea, which was performed in FinnGen (Round 8).[3]

Table 1: Included outcome GWAS for colocalization analyses

MRC-IEU GWAS ID	Outcome	Number of cases	Number of Controls	year	consortium
ukb-b-18700	cholelithiasis/gallstones (self-report)	7682	455251	2018	UK Biobank
ieu-b-4971	Cholecystitis (ICD-10 coded)	4052	482432	2021	UK Biobank
ukb-b-13803	Cholecystectomy (OPCS coded)	10361	452649	2018	UK Biobank
N/A	Pityriasis Rosea (ICD-10 coded)	143	**299128**	2022	FinnGen (R8)

In colocalization, we found reasonable evidence that these outcomes colocalised with bilirubin levels at this loci (H_4 for cholelithiasis 0.61; H_4 for cholecystitis 0.66; H_4 for cholecystectomy 0.65; H_4 for pityriasis rosea 0.15). The lower posterior probability of a shared causal variant for pityriasis rosea likely reflects the lower power in this GWAS (n cases = 143). Sensitivity plots for pityriasis rosea are available in **Figure 1,** with full details and replicable code for other outcomes available via the data availability section of the manuscript and at

Figure 1: Effect of varying the prior probability of a shared causal variant (p12) on the posterior probability of a shared causal variant for bilirubin and pityriasis rosea. This shows that the posterior probability of a shared causal variant requires a reasonably high prior probability of a shared causal variant.

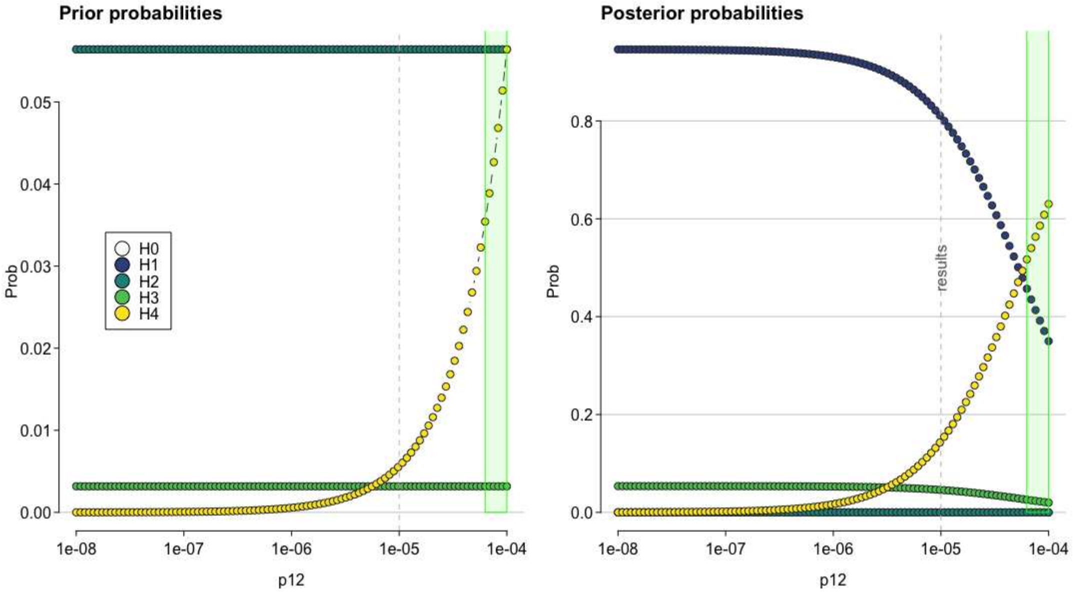

1. Giambartolomei C, Vukcevic D, Schadt EE, et al. Bayesian test for colocalisation between pairs of genetic association studies using summary statistics. PLoS Genet [Internet] 2014;10(5):e1004383. Available from: http://dx.doi.org/10.1371/journal.pgen.1004383

2. Pan UKBB [Internet]. [cited 2022 Oct 13];Available from: https://pan.ukbb.broadinstitute.org/

3. Kurki MI, Karjalainen J, Palta P, et al. FinnGen: Unique genetic insights from combining isolated population and national health register data [Internet]. bioRxiv. 2022 [cited 2022 Apr 7];2022.03.03.22271360. Available from: https://www.medrxiv.org/content/10.1101/2022.03.03.22271360v1.abstract
