# Supplementary material for: Effect of bilirubin and Gilbert syndrome on health: cohort analysis of observational, genetic, and Mendelian randomisation associations
Source: BMJ Med. 2023 Jul 12;2(1):e000467. doi: 10.1136/bmjmed-2022-000467 (PMC10347488; doi:10.1136/bmjmed-2022-000467)
Supplement: Supplementary data [file bmjmed-2022-000467supp001.pdf]

Supplementary Figures

Supplementary Figure S1: Power calculations for our genetic analysis. Power was calculated as per[1], using an alpha of 0.05, and assuming an instrumental variable with an R^2 of 0.37. Plot A shows the change in power with increasing sample size of cases (total N fixed at 450,000) for a variety of odds ratios, while plot B shows the change in power with increasing odds ratios for a variety of case counts.

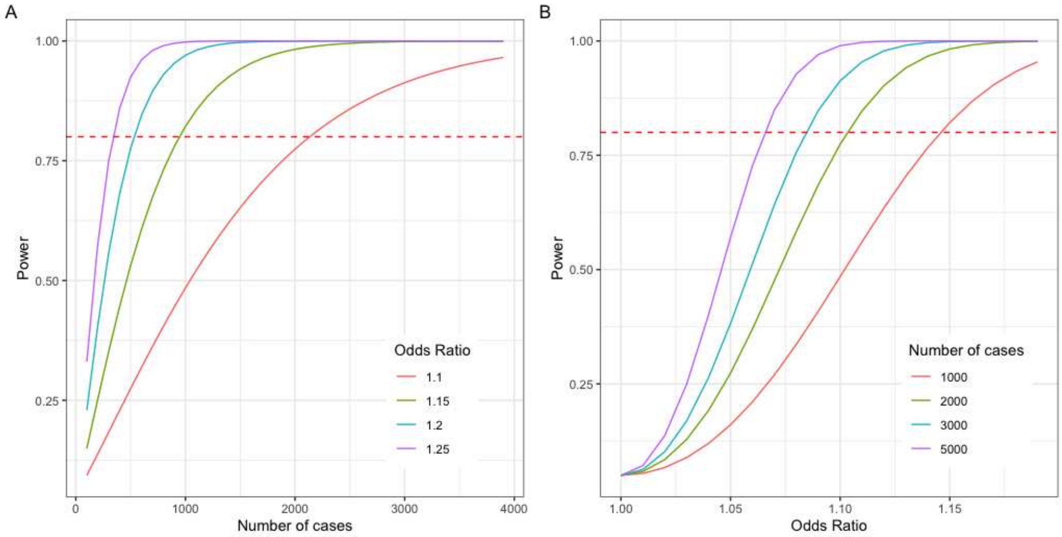

Supplementary Figure S2: Associations between estimates in men (x axis), and women (y axis) for carriage of the Gilbert's syndrome genotype for all associations that met a nominal (p <0.05) association in sex-combined analyses.

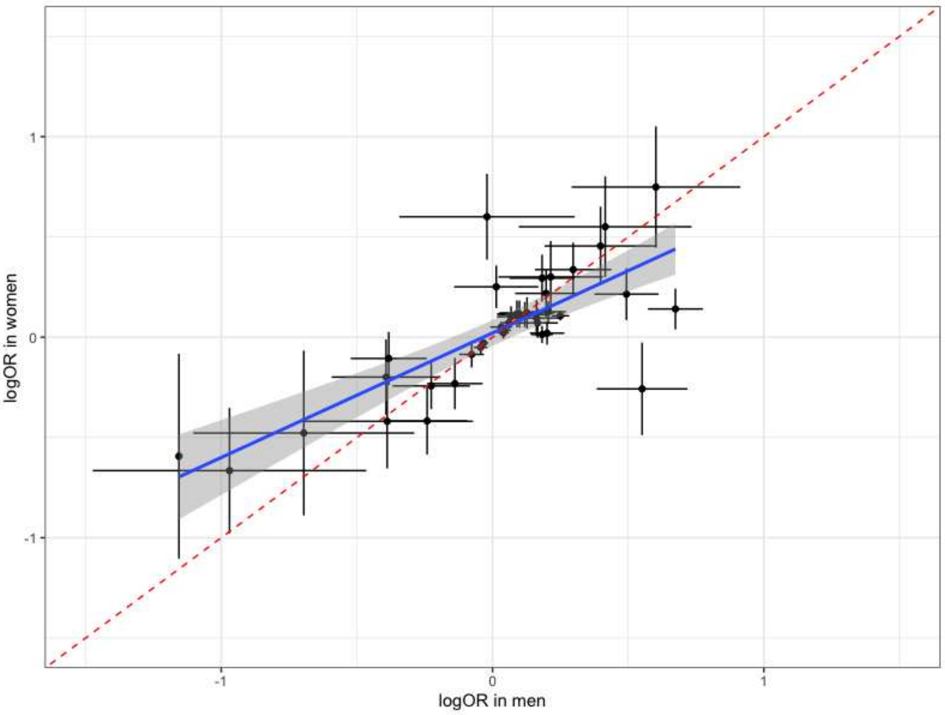

Supplementary Figure S3: Forest plot of associations between Gilbert's syndrome genotype and each outcome, stratified by sex. This forest plot only shows those associations that had a nominal (p < 0.05) heterogeneity across sexes.

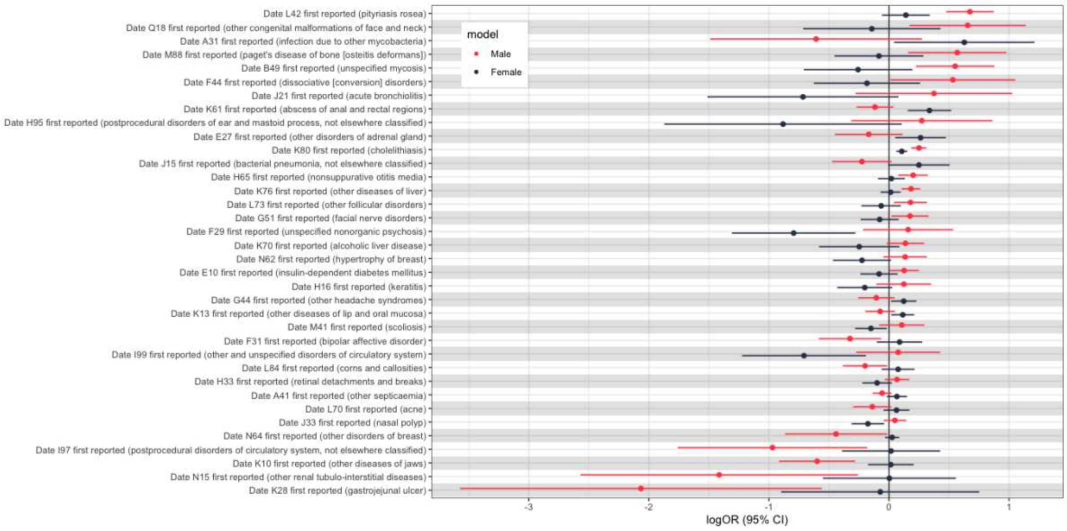

1. Brion M-JA, Shakhbazov K, Visscher PM. Calculating statistical power in Mendelian randomization studies. Int J Epidemiol [Internet] 2013;42(5):1497–501. Available from: http://dx.doi.org/10.1093/ije/dyt179
